# Urban methane emission monitoring across North America using TROPOMI data: an analytical inversion approach

**DOI:** 10.1038/s41598-024-58995-8

**Published:** 2024-04-19

**Authors:** Mohammadali Hemati, Masoud Mahdianpari, Ray Nassar, Hodjat Shiri, Fariba Mohammadimanesh

**Affiliations:** 1 Department of Electrical and Computer Engineering, Faculty of Engineering and Applied Sciences, Memorial University of Newfoundland, St. John’s, Canada; 2 C-CORE, 1 Morrissey Road, St. John’s, NL Canada; 3 Climate Research Division, Environment and Climate Change Canada, Toronto, ON Canada; 4 Civil Engineering Department, Faculty of Engineering and Applied Sciences, Memorial University of Newfoundland, St. John’s, Canada

**Keywords:** TROPOMI, Atmospheric inversion, Methane emission, Urban methane monitoring, North America, GHG, Atmospheric chemistry, Electrical and electronic engineering

## Abstract

Monitoring methane emissions is crucial in mitigating climate change as it has a relatively short atmospheric lifetime of about 12 years and a significant radiative forcing impact. To measure the impact of methane-controlling policies and techniques, a deep understanding of methane emissions is of great importance. Remote sensing offers scalable approaches for monitoring methane emissions at various scales, from point-source high-resolution monitoring to regional and global estimates. The TROPOMI satellite instrument provides daily XCH_4_ data globally, offering the opportunity to monitor methane at a moderate spatial resolution with an acceptable level of sensitivity. To infer emissions from TROPOMI data, we used the prior emission estimates from global and national inventories and the GEOS-Chem chemical transport model to simulate atmospheric methane along with actual observations of TROPOMI. In this study, methane emissions from Toronto, Montreal, New York, Los Angeles, Houston, and Mexico City have been estimated using the analytical solution of Bayesian inversion using the cloud-based Integrated Methane Inversion (IMI) framework. Using the result from ensemble inversions, and city boundaries, the average total emissions were as follows: Toronto 230.52 Gg a^−1^, Montreal 111.54 Gg a^−1^, New York 144.38 Gg a^−1^, Los Angeles 207.03 Gg a^−1^, Houston 650.16 Gg a^−1^, and Mexico City 280.81 Gg a^−1^. The resulting gridded scale factors ranged from 0.22 to 6.2, implying methane prior emission underestimations in most of these cities. As such, this study underscores the key role of remote sensing in accurately assessing urban methane emissions, informing essential climate mitigation efforts.

## Introduction

Methane (CH_4_) is the second most important greenhouse gas with a relatively short average atmospheric residence period (i.e., twelve years) compared to carbon dioxide (CO_2_)^1^. As a consequence of anthropogenic activities, methane has continued to accumulate in the atmosphere since pre-industrial times and has directly contributed to 0.6 °C of global warming^2^. Including impacts on water vapor in the stratosphere and tropospheric ozone, the methane emission-based radiative forcing is about 0.97 Wm^−2^. Since the pre-industrial time, this radiative emission forcing has been almost 60% of that carbon dioxide^3^.

Anthropogenic and human-related methane emissions are from waste management, livestock, coal mining, rice cultivation, and energy-related industries^4^. Globally, it is estimated that 100 to 180 Tg a^−1^ of anthropogenic methane emissions are from oil, gas, and other fossil fuel energy sectors, which is almost a third of all contemporary human emissions^5–8^. A major focus of climate policies is controlling methane emissions from anthropogenic sectors. As such, methane-related controlling policies are regarded as essential for slowing the pace of global temperature change in the near future^9^. Enhanced comprehension of emissions processes and the capacity to evaluate the effects of these policies and techniques are crucial for the effective mitigation of greenhouse gases, including methane, and their impact on climate change^10^. Since 1950, the population of humankind living in cities has increased significantly from 751 million people to 4.2 billion in 2018^11^. This rapid urbanization changed global energy and resource usage patterns, which affects the environment and climate enormously^12,13^. However, methane emissions from metropolitan areas across the world are poorly known at present, and this can stand as a possibly significant source of CH_4_ to be targeted for near-term mitigation^14^. Previous studies were often based on direct local measurement of methane by mobile platforms through sporadic sampling or installation of a network of instruments (e.g., towers) and were mostly limited to specific urban regions^15–17^.

For moving from point source to urban-scale or regional scale, remote sensing provides the spatial and spectral requirements to monitor methane emissions from both anthropogenic and natural sources^18^. Atmospheric methane column concentrations are measured by earth observation platforms with shortwave infrared (SWIR) or thermal infrared (TIR) spectral bands. The effectiveness of SWIR-derived methane concentration by earlier instruments, such as SCanning Imaging Absorption spectroMeter for Atmospheric CHartographY (SCIAMACHY) and Greenhouse gases Observing SATellite (GOSAT) has been demonstrated in the literature^19–21^ since these observations offer sensitivity to CH_4_ near Earth’s surface. However, sparse sampling or coarse pixel resolution were the main limitations of these missions, which makes their application limited and challenging at the metropolitan scale. In October 2017, Copernicus and the European Space Agency (ESA) launched the TROPOspheric Monitoring Instrument (TROPOMI) on board the Sentinel-5 Precursor satellite. This instrument provides daily observations of parameters relating to clouds and aerosols, in addition to observations of carbon monoxide, methane, ozone, sulfur dioxide, nitrogen dioxide, and formaldehyde^22^. With a daily overpass around 13:30 local time, reflected sunlight is measured by the onboard spectrometer in different spectral bands (i.e., Shortwave Infrared, Near Infrared, Visible, and Ultraviolet). TROPOMI Level-2 CH_4_ data was initially available with 7 × 7 km^2^ spatial resolution since early 2018, but resolution improved to 5.5 × 7 km^2^ in August 2019^23^. The near-global coverage of TROPOMI compared to the sparse coverage of GOSAT, along with its higher spatial resolution in comparison with previous instruments (e.g., 60 × 30 km^2^ for SCIAMACHY and 10.5 km diameter for GOSAT), provides unprecedented capability for detection and quantification of methane emissions^23^.

Under the United Nations Framework Convention on Climate Change (UNFCCC), countries are required to report their anthropogenic methane emissions using the accepted guidelines of the Intergovernmental Panel on Climate Change (IPCC) Task Force on Emission Inventories (TFI), last updated in 2019^24^. Emission factors and activity data, such as emissions per head of cattle, are used to create bottom-up emission reports. To evaluate bottom-up methane inventories, Earth observation data can be used through different approaches, such as point source plume detection^25^, CH_4_ and CO_2_ ratio^26^, mass balance, and inverse modeling^27^, among other methods. Numerous studies found higher anthropogenic emissions than reported by multiple different countries. Using GOSAT observations between 2009 and 2012, a previous study estimated that anthropogenic methane emissions over North America were 28% higher than the reported amount^28^. Moreover, studies focused on the United States quantified higher emissions than the Environmental Protection Agency (EPA) reports^29^. These studies suggested up to 50% higher emissions attributed to fossil fuels (oil and gas)^30^ and livestock emission^21^. Also, the contribution of North American emissions to climate change and the increasing global methane trend has been the subject of substantial debate. In a study using GOSAT observations between 2010 and 2016, an annual 2.5 ± 1.4% increase in the U.S. has been estimated and attributed to oil and gas activity, interannual variation in Canada because of wetlands, and a 0.8 to 1.4% decrease in Mexico for livestock^31^.

Urban methane monitoring has become demanding, especially in recent years. Dedicated to climate change mitigation, C40, a performance-oriented coalition involving over 100 mayors, is recommending that cities work towards a 50% reduction in methane emissions by the year 2030^32^. Cities have the potential to effectively mitigate methane emissions by executing leak-detection programs, waste-reduction initiatives, and fostering strategic partnerships with gas utilities and landfill operators. Moreover, regulatory frameworks established by air pollution control districts can significantly enhance urban efforts to reduce emissions^14^. In some studies, in-situ measurements from towers^33–36^ or airborne observations were used to measure methane emission^37–40^. Different methods, such as mass balance^38,41^ or ratio (for CO_2_ or CH_4_)^42,43^, along with the inversion^44,45^ were used in previous urban studies. For monitoring methane emission over metropolitan areas (i.e., urban scale), previous studies also used TROPOMI data along with carbon monoxide (CO) retrieval and coincident observations of multiple species^26,46^. In a more advanced national study, TROPOMI data was used to map urban emissions using the analytical inversion^32^. Studies using in-situ or airborne measurements require resources (such as measuring towers or aircraft) and also lack the spatial coverage of satellite observations. Furthermore, using satellite observations for methane emission requires computational resources to store and compute massive amounts of data. Therefore, in this study, the application of the recently developed cloud-computing platform, called Integrated Methane Inversion (IMI)^47^, for measuring methane emission on an urban scale was investigated.

Accordingly, the main objective of this study can be summarized as follows:

- Mapping urban methane emission across some of the biggest North American metropolitan areas.
- Leveraging a cloud computing platform and TROPOMI data, along with Bayesian inverse analysis for the application of urban methane emission.
- Quantifying methane emissions in different cities and analyzing the urban emission patterns.

Inverse analyses seek to optimize the estimation of methane emissions, represented as the state vector, by aligning observed TROPOMI data with simulated concentrations generated by a Chemical Transport Model (CTM), serving as the inversion forward model. This optimization process usually involves minimizing a Bayesian cost function regularized by a prior emission derived from a bottom-up inventory. In instances where a linear connection exists between emissions and concentrations, such as with methane, it becomes possible to analytically determine the optimal (posterior) solution, along with the corresponding error covariances^48^. In this study, IMI^47^ was used to access TROPOMI data, along with GEOS-Chem CTM and national or global bottom-up inventories, to create an ensemble of inversions to analyze methane emissions over six cities in North America.

## Results and discussion

**Figure 1:**
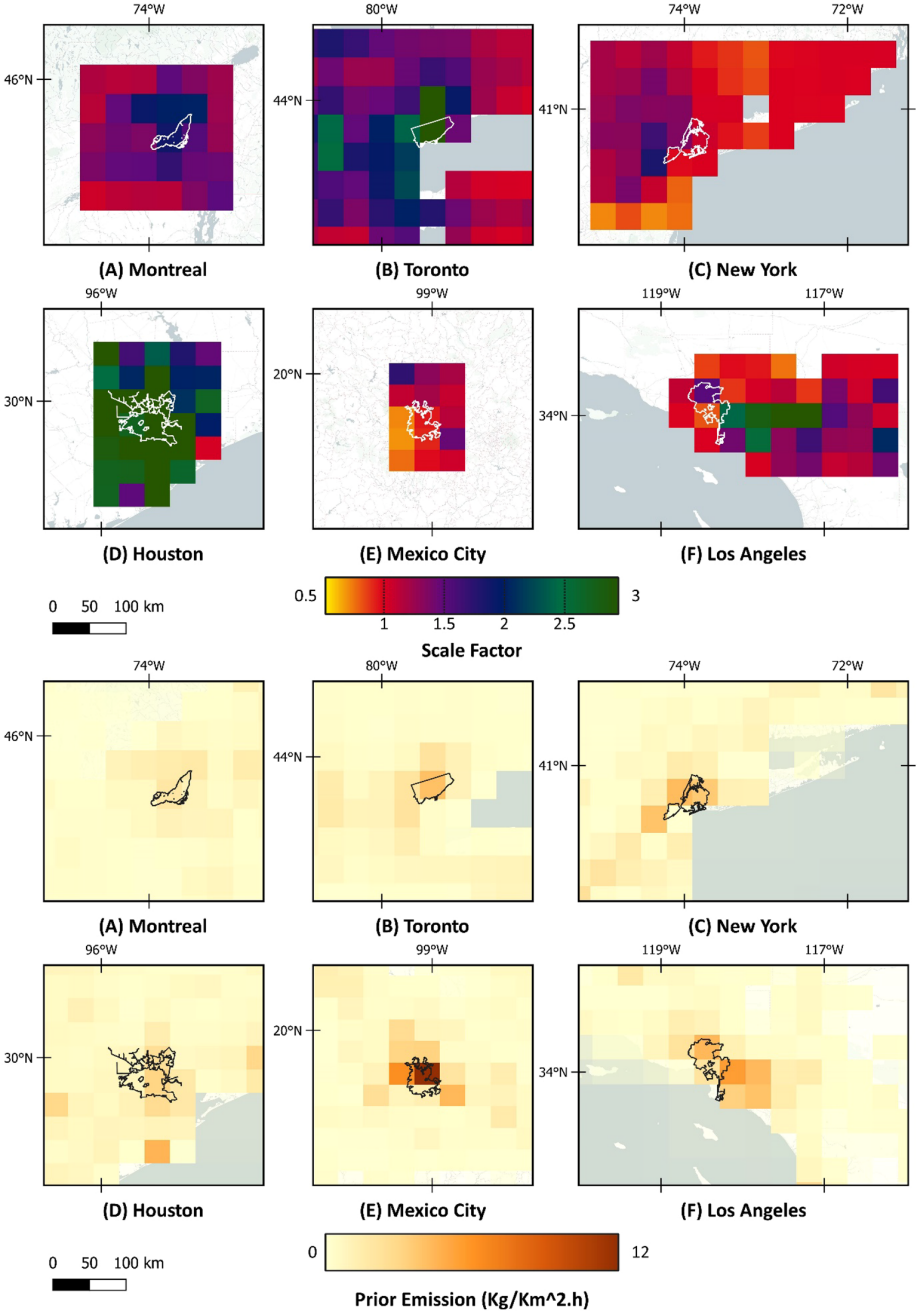
Scale factor (Posterior/Prior emission), derived from inversion results, and the total prior emission are shown at the native resolution of the model. Map was created using open-source QGIS 3.34.2 (https://qgis.org/en/site/).

The feasibility of applying Sentinel-5P TROPOMI daily observation of methane concentration for monitoring urban-scale methane emissions using the IMI^47^ cloud-based computing platform was investigated in this study. Methane concentration from 2021 was used together with the GEOS-Chem forward model to solve the Bayesian inverse problem. Using the TROPOMI data, methane emissions in six North American metropolitan areas, including Toronto, Montreal, New York, Los Angeles, Houston, and Mexico City, have been quantified through an analytical inversion technique. The bottom-up inventories, accessed from the IMI platform^47^, created the prior emission for the inversion (Table 3). The anthropogenic sources that were used in the prior emission were the spatially-distributed gridded inventories of: (A) The U.S. emissions based on the Environmental Protection Agency (EPA) Greenhouse Gas Inventory (GHGI) Inventory of US Greenhouse Gas Emissions and Sinks^49^ for 2012, (B) Canadian emissions based on the Environment and Climate Change Canada (ECCC) National Inventory Report for Canada^50^ for 2018, and (C) Mexico’s emissions based on the National Institute of Ecology and Climate Change (INECC) national inventory^51^ for 2015. Figure 1 illustrates the scale factor values obtained from solving an inversion problem and the total prior emission in the region of interest.

**Figure 2:**
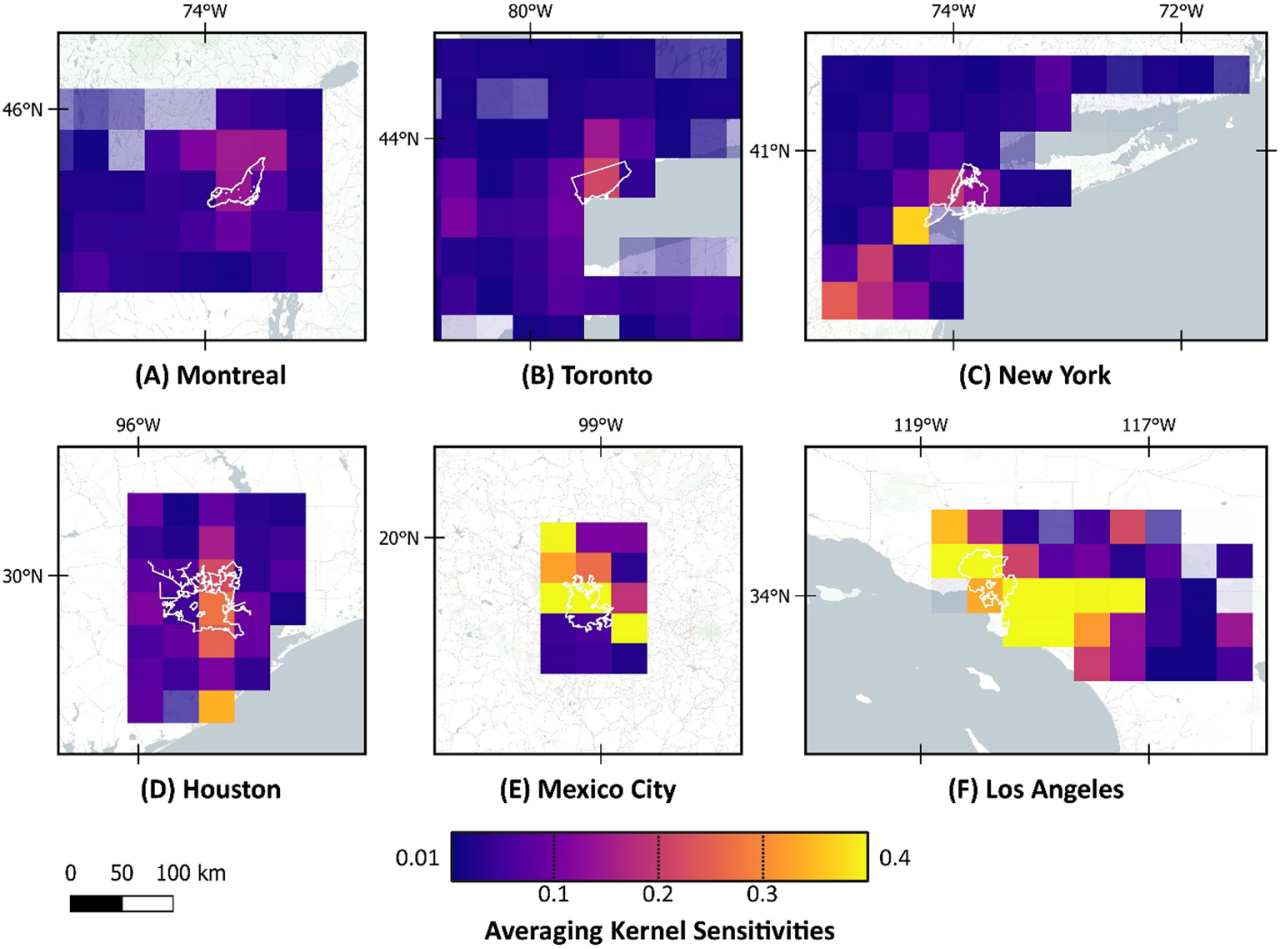
AKS of the inversion results. Map was created using open-source QGIS 3.34.2 (https://qgis.org/en/site/).

**Figure 3:**
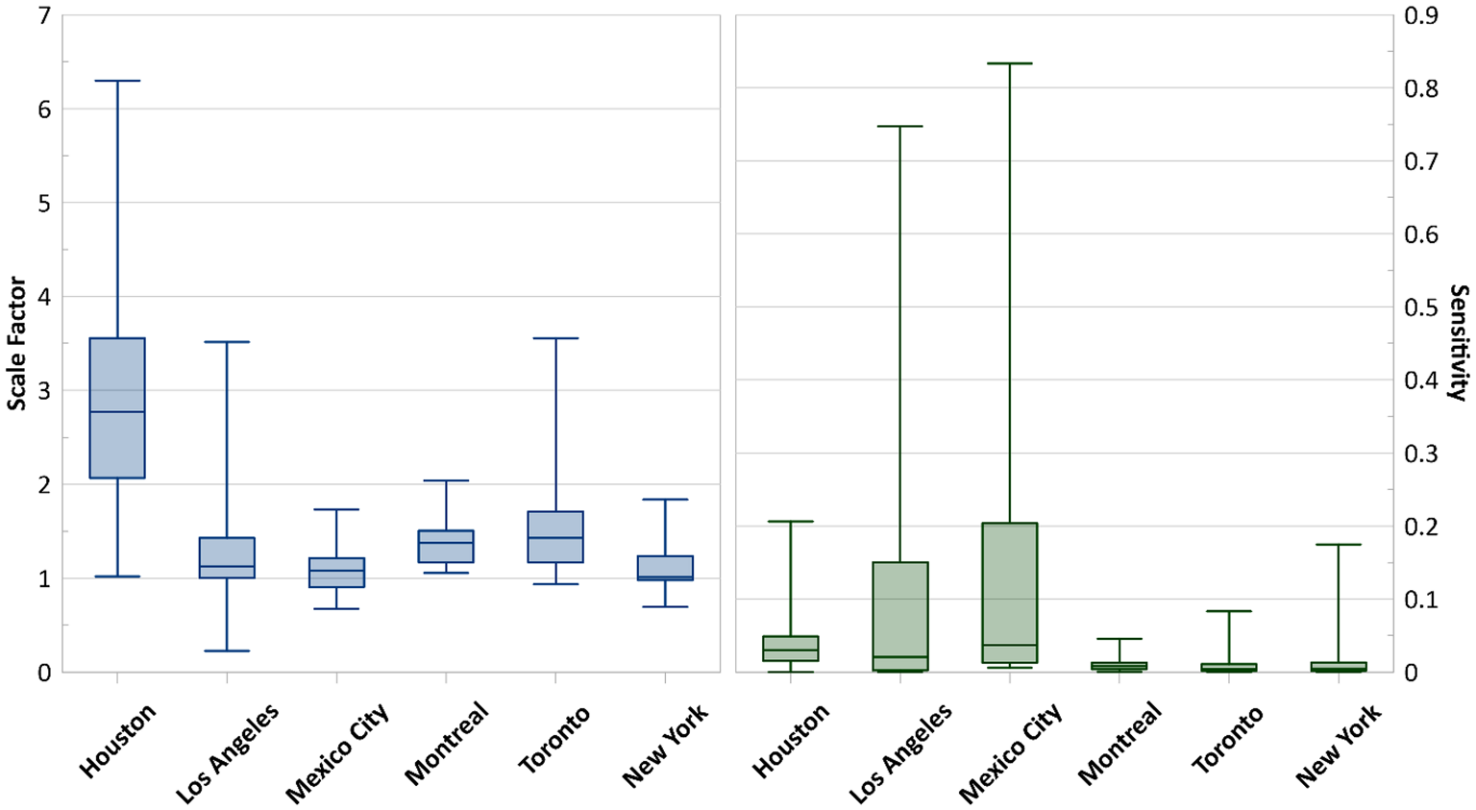
The scale factor and sensitivity in different cities.

A correction scale factor varying between 0.22 and 6.2 was applied to prior emissions in the study area, illustrating an underestimation of the prior methane emission in most study areas. In New York, Montreal, and Mexico City, the scale factor was under 2. However, in Los Angeles and Toronto, the scale factor was raised to 3.5 in central regions and was significantly high in the Houston area. In Los Angeles, Mexico City, New York, and Toronto, there were limited areas where the scale factor was below 1, mostly in suburban areas, meaning the prior inventory was overestimating the methane emission in those regions. The Averaging Kernel Sensitivity (AKS) map of the different metropolitan areas is illustrated in Fig. 2. AKS simply shows the information content for each element of inversion (state vector). Figure 3 shows the scale factor range and sensitivity in each city. The sensitivity of the posterior estimate is assessed based on the observations, as diagnosed through the averaging kernel matrix. This sensitivity metric spans a range from 0, indicating no sensitivity, and the posterior being equal to the prior, to 1, signifying full sensitivity, where the posterior is solely determined by the observations.

An ensemble inversion has been applied to find the sensitivity of the inverse model to its parameters, including the regularization parameter (γ) and prior error (PE). After calculating the Jacobian matrix, different inversion results have been produced using the parameters in Table 1. Based on the default parameters, as well as the suggested values in previous studies using TROPOMI data^1,47^, a grid search was implemented to create ensemble members and different scenarios. The regularization parameter plays a crucial role in preventing overfitting or underfitting caused by imprecise specifications of prior and observation covariance matrices. In this context, increasing the regularization parameter to 1 means that all observations are independent.

**Table 1:** The result of the inversion model with different parameters.

Toronto			Montreal			Los Angeles			Houston			New York			Mexico City		
γ	PE	DOF	γ	PE	DOF	γ	PE	DOF	γ	PE	DOF	γ	PE	DOF	γ	PE	DOF
0.10	50	0.27	0.10	50	0.20	0.10	50	3.20	0.10	50	0.65	0.10	50	0.34	0.10	50	1.38
0.25	50	0.65	0.25	50	0.47	0.25	50	4.92	0.25	50	1.41	0.25	50	0.79	0.25	50	2.10
0.50	50	1.22	0.50	50	0.90	0.50	50	6.56	0.50	50	2.38	0.50	50	1.42	0.50	50	2.86
0.25	70	1.20	0.25	70	0.88	0.25	70	6.51	0.25	70	2.34	0.25	70	1.40	0.25	70	2.84
0.50	70	2.20	0.50	70	1.64	0.50	70	8.50	0.50	70	3.77	0.50	70	2.39	0.50	70	3.80
0.75	90	4.59	0.75	90	3.44	0.75	90	11.60	0.75	90	5.88	0.75	90	4.46	0.75	90	5.00
1.00	100	6.63	1.00	100	4.97	1.00	100	13.50	1.00	100	8.52	1.00	100	6.06	1.00	100	6.27

To improve the degrees of freedom (DOF), the length of the inversion can be increased with more observation, which can be costly using cloud platforms. Also, a higher-quality bottom-up inventory with more accurate methane emission estimation can be used to enhance the inversion results. However, in Table 1, the posterior result with the highest DOF may not be the best result since it may be the result of an overfitted model, which is challenging to identify on an urban scale. To include the errors of inversion parameters, including the regularization parameter and prior inventory error covariance matrices, the average results were reported. In this way, the ensemble range highlights the uncertainty ranges.

**Figure 4:**
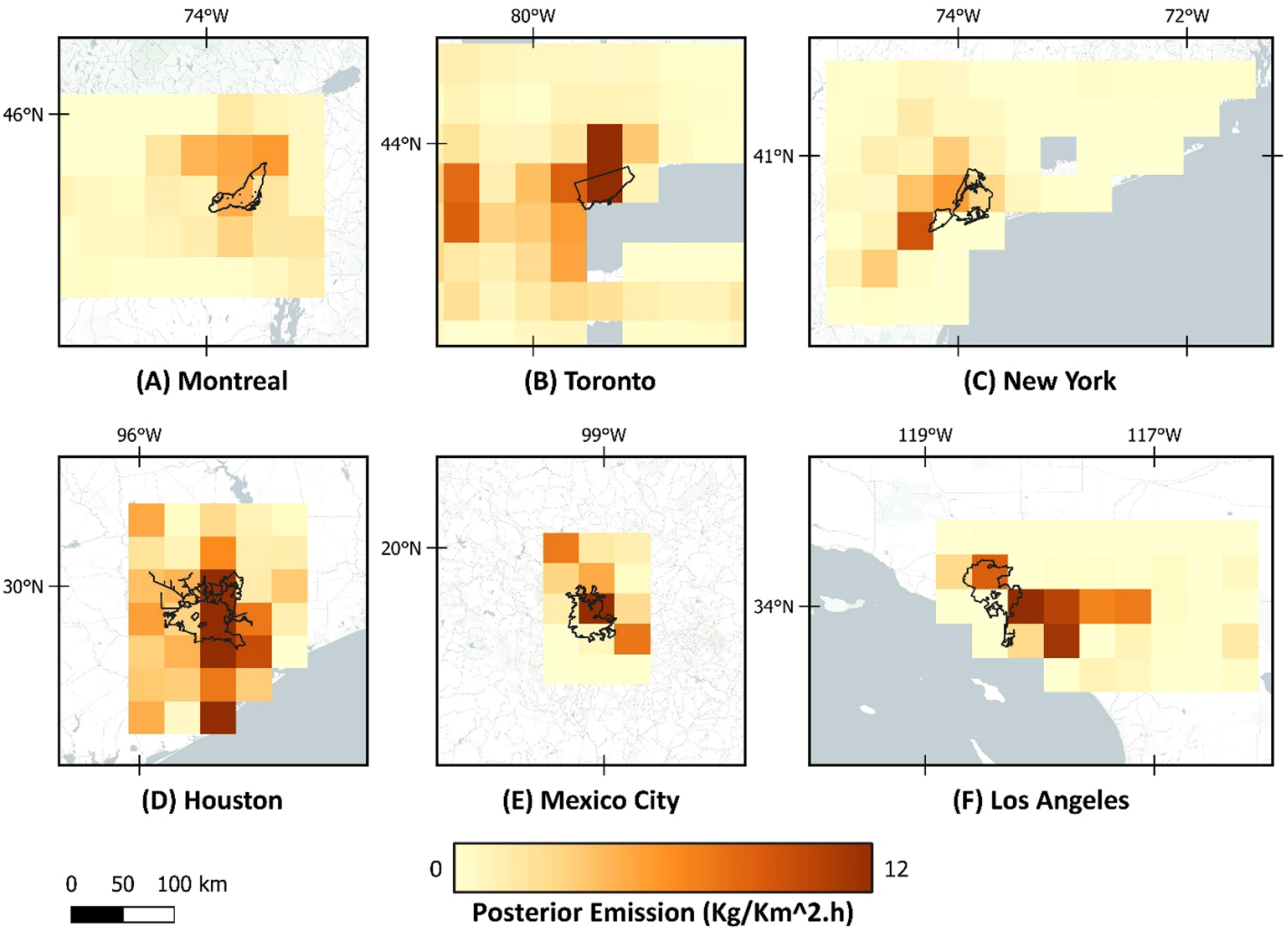
Posterior estimation of methane emissions, based on atmospheric analysis of TROPOMI data in 2021. Map was created using open-source QGIS 3.34.2 (https://qgis.org/en/site/).

The quantification of methane emissions from prior sectors and posterior total emission is reported in Table 2. The quantification is based on the official city boundaries shown with the black line in Fig. 4, explained in “Study area”. For Toronto and Montreal, the prior oil and gas emissions from the bottom-up inventory were reported together^50^. The uncertainties of each ensemble member are reflected through the posterior error covariance matrix and the averaging kernel sensitivities. The error bars for methane emissions were calculated using the ensemble values to reflect the errors from inversion parameters and uncertainty range. Considering the official city boundaries for urban areas, average total emissions quantified and the ensemble range for cities are as follows: Toronto 230.52 (110.11–327.33) Gg a^−1^, Montreal 111.54 (60.97–172.61) Gg a^−1^, New York 144.38 (116.62–161.46) Gg a^−1^, Los Angeles 207.03 (197.30–237.17) Gg a^−1^, Houston 650.16 (342.01–806.66) Gg a^−1^, and Mexico City 280.81 (250.37–334.70) Gg a^−1^. Emission values obtained in this study are also shown in Fig. 4. As seen, there are methane emission hotspots in the centers of Toronto, Mexico City, and Houston.

**Table 2:** Prior emissions from different sectors, along with total prior and average posterior emission (Gg a^−1^).

	Toronto	Montreal	New York	Los Angeles	Houston	Mexico City
Biomass burn	0.00	0.00	0.00	6.9E−04	8.20E−04	1.14
Coal	0.00	0.00	0.00	0.00	0.00	0.17
Oil	10.72	4.14	0.09	26.13	10.33	0.17
Gas			32.80	17.15	28.11	3.45
Landfills	58.10	46.39	52.36	101.30	66.88	262.76
Livestock	0.24	1.57	0.12	0.77	7.80	13.51
Other Anthropogenic	4.82	4.03	13.04	8.15	7.48	18.47
Rice	0.00	0.00	0.00	0.00	0.63	0.00
Seeps	0.03	0.00	0.00	2.29	1.48	0.06
Soil absorbtion	− 0.06	− 0.02	− 0.38	− 0.76	− 1.39	− 3.53
Termites	0.24	0.32	0.49	0.11	0.59	0.27
Waste water	3.44	2.01	24.13	7.81	5.46	36.96
Wetlands	4.75	2.28	7.03	1.58	4.84	0.11
Prior total	82.28	60.72	129.67	164.53	132.22	333.53
Average posterior total	230.52	111.54	144.38	207.03	650.16	280.81

**Figure 5:**
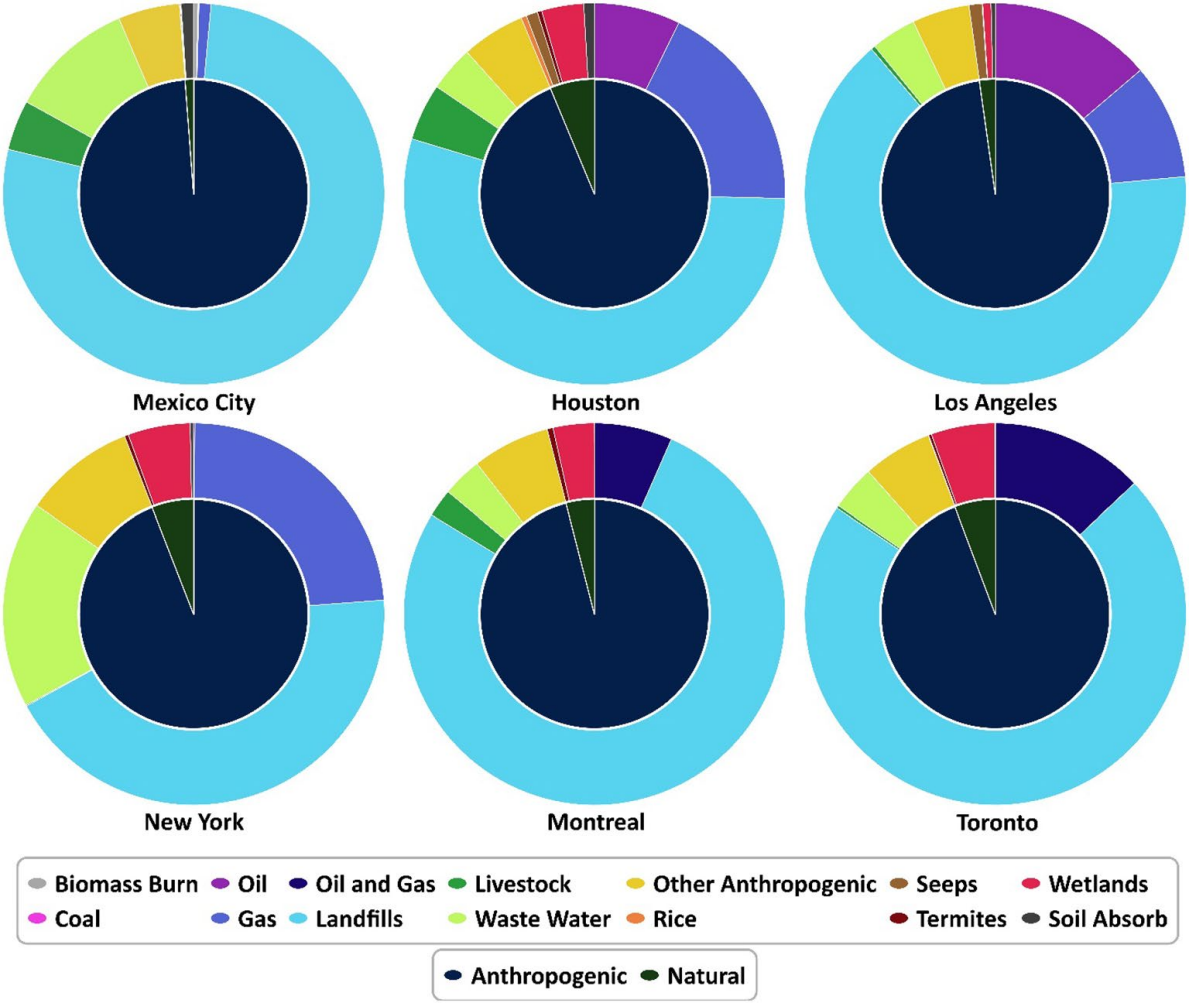
Contribution of each sector to total prior methane emission in different cities.

Due to the low resolution of the IMI results and co-location of different sources, the posterior results from different sectors are not reported. Sectoral estimates are also more uncertain than the total methane emissions, and the sectoral uncertainties are difficult to quantify. Figure 5 illustrates the relative contributions of various sectors to atmospheric methane emission from prior inventories. Each sector's contribution is depicted as a proportion of the total prior methane emission, providing a visual representation of their respective impact on atmospheric methane. This information can be valuable for policymakers and stakeholders seeking to prioritize interventions and strategies to mitigate methane emissions. As seen, a substantial portion of methane emissions in all six metropolitan areas can be attributed to human activities, with anthropogenic sources accounting for a significant share of the total emissions. It is crucial to identify and address these anthropogenic sources to effectively reduce methane emissions and mitigate their impact on the environment and climate. All the used anthropogenic methane inventories from the IMI, including all the emitters from urban areas, are at 0.1° × 0.1° (~ 10 × 10 km^2^) spatial resolution^47^. TROPOMI methane retrieval is also at 7 × 5.5 km^2^ spatial resolution after August 2019. However, due to the coarse spatial resolution of meteorological data, the posterior result is at 0.25° × 0.3125° (~ 25 × 30 km^2^). This is the nested, high-resolution version of the GEOS-Chem, yet unsuitable for monitoring methane at the facility level in dense areas, such as urban areas, where sources are co-located. The method employed in this study demonstrates the feasibility of utilizing open-access TROPOMI observations and the IMI cloud platform^47^ to effectively monitor total methane emissions in urban areas. This approach provides a cost-effective and scalable means of monitoring emissions at the city or regional level, which can inform targeted mitigation efforts and help reduce the overall impact of methane emissions on climate change. The successful application of this method also underscores the potential of satellite-based observations for supporting global efforts to track and reduce greenhouse gas emissions. TROPOMI data can be used to monitor individual sectors and emitters when they are not co-located with other sectors^52^. However, higher-resolution observations provide more detailed information for monitoring and measuring methane emissions from individual sectors and facilities (point-source emissions). For example, the feasibility of targeted high spatial resolution satellite observations (e.g., GHGSat, MethaneSat) along with TROPOMI data has been assessed for monitoring methane emissions in landfills^53^.

**Figure 6:**
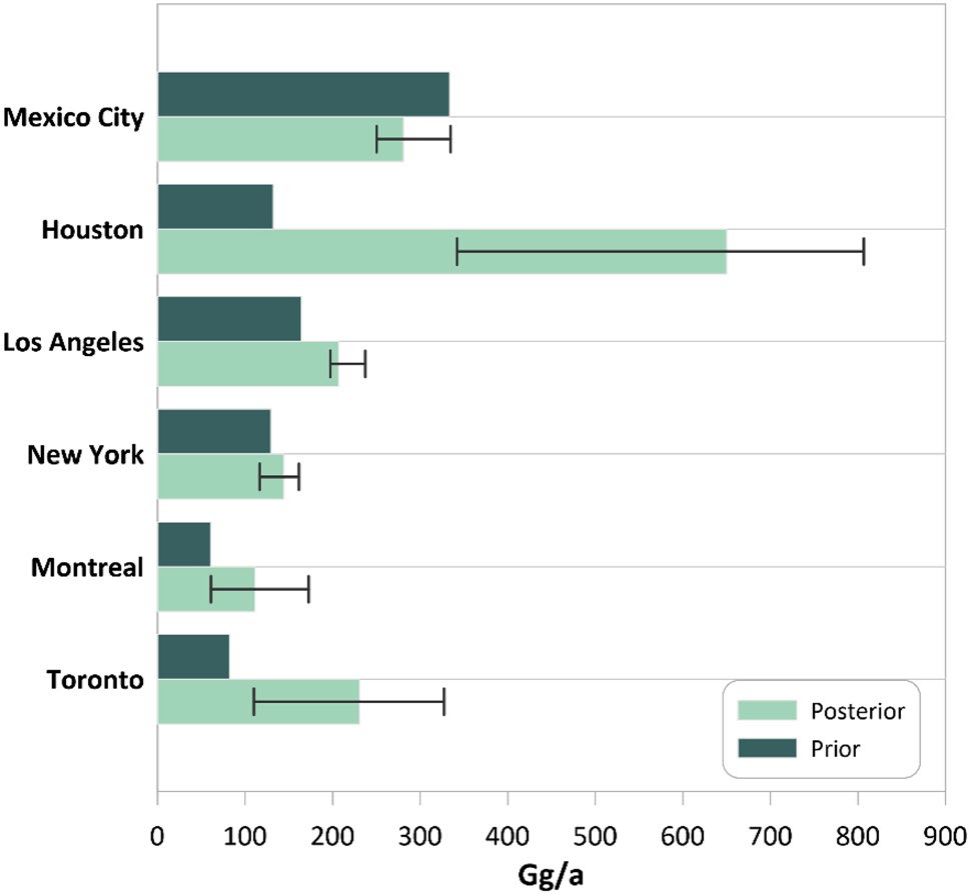
The estimated total amount of prior and posterior emission. The spread of ensemble results is shown as error bars.

The estimated posterior emission was also compared to prior bottom-up methane inventories in Fig. 6. In most metropolitan areas, the estimated emissions were higher than the bottom-up inventory. In Mexico City, the estimated emission was a bit lower than the prior emission, showing the overestimation of emissions from this city. The discrepancy between the prior and posterior emissions was relatively small in Montreal, New York, and Los Angeles but substantial in Toronto, especially Houston.

Comparison of the quantified values for total urban emissions with previous studies can be challenging due to the different boundaries considered for cities and the estimated emissions for different years. Total emissions estimated for New York are consistent with previous studies conducted in that metropolitan area^26^. The estimated 144.38 Gg a^−1^ is comparable with the inventory reported by the city of New York^32^, which is just above 100 Gg a^−1^. Considering a larger boundary for New York, New Jersey, and Connecticut, a total emission of 309 Gg a^−1^ was estimated using TROPOMI^32^, and 314 Gg a^−1^ using aircraft data^37^ was reported in recent studies. In this study, Los Angeles emission was quantified at 207.03 Gg a^−1^, which is close to previous studies^41^. For Los Angeles, many studies estimated around 400 Gg a^−1^ methane emission for larger metropolitan area^33,34,42,43^. Another recent study estimated Los Angeles methane emission to be 121 Gg a^−1^ and found that it is overestimated^32^. Moreover, in another study, the declining trend in Los Angeles from 2015 to 2020 was found^54^, with emission of 251 Gg a^−1^ in 2019. For Toronto, estimated CH_4_ emissions have some inconsistencies with reported emissions under Canada’s facility-level Greenhouse Gas Reporting Program (GHGRP). For 2021, the GHGRP includes gas distribution emissions of 17.1 Gg a^−1^ and gas pipeline transmission emissions of 5.7 Gg a^−1^ within Toronto, which together are roughly equal to the total estimated emissions of Toronto. The lower estimate derived from this study is attributed to the lower estimate in the prior, which used reported data from 2018 (9.5 Gg a^−1^ for gas distribution within the City of Toronto). However, by optimizing the parameters of the inversion, the prior emission error can be controlled. The GHGRP also reports CH_4_ emissions from 4 large landfills (Keele Valley, Brittania, Beare Road, Thackeray) surrounding the city of Toronto, which equals 33.2 Gg a^−1^. Although none of these are in the city proper, they would be difficult to distinguish at the resolution of this study. Lower emissions from the Keele Valley landfill than reported would be consistent with recent urban CH_4_ inversions using mobile in situ data^55^ and the overall reduction of GTA landfill emissions for a GTA inventory^56^, compared with the GHGRP.

**Figure 7:**
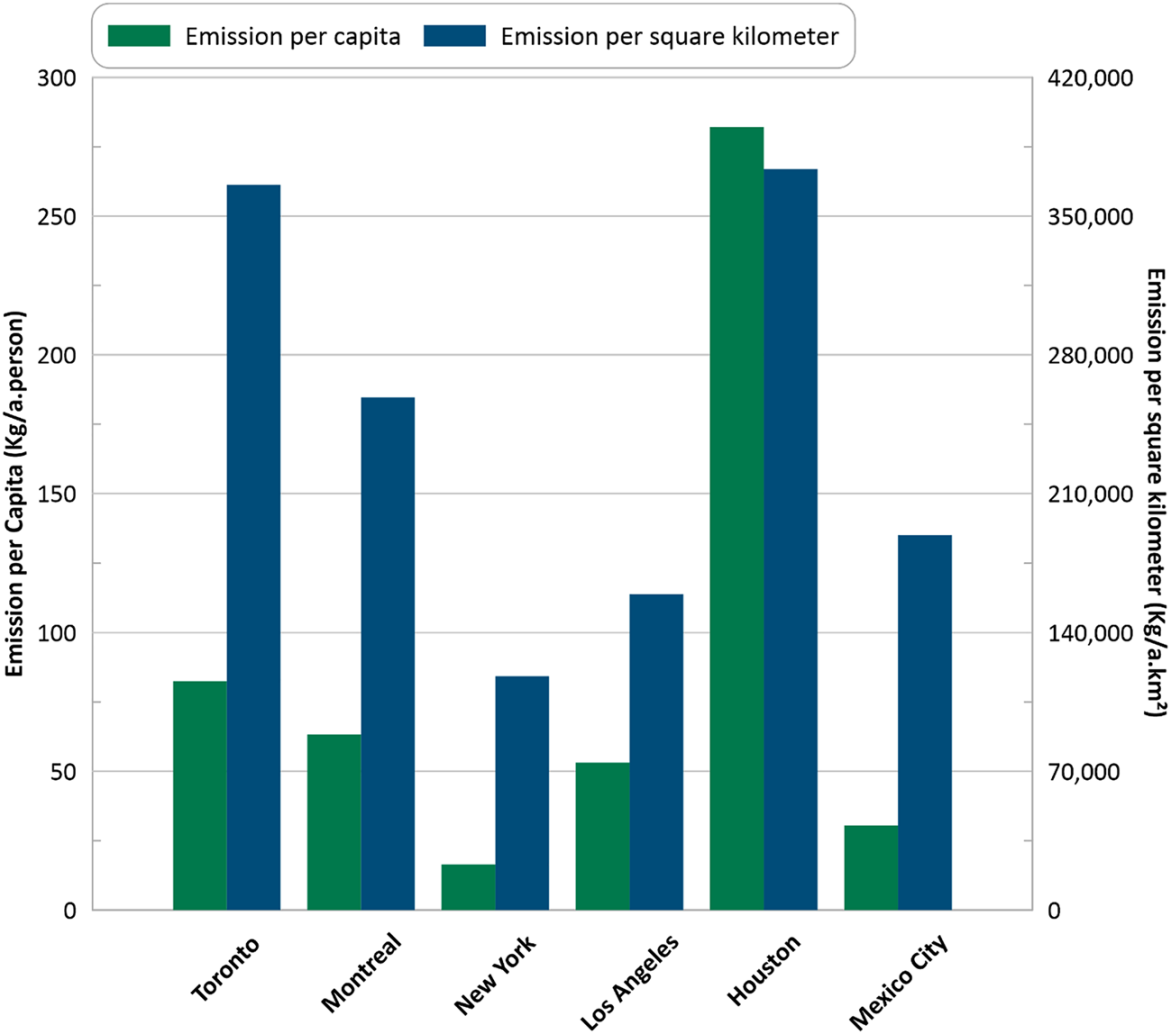
Normalized amount of methane emission per capita and per square kilometer.

Figure 7 presents a normalized comparison of methane emissions in the six metropolitan areas based on both population and area. Notably, Houston exhibited significantly higher emissions per capita and per square kilometer compared to other cities, which can presumably be attributed to its oil and gas industry. Among the other cities, Toronto had a higher emission per area than other cities, but similar per capita emissions compared to Montreal and Los Angeles. In contrast, New York had the lowest emission per capita, followed by Mexico City. By providing a normalized comparison of methane emissions, this analysis highlights the variation in emissions across different cities and can help identify areas that may require targeted mitigation efforts to reduce the impact of methane on the environment and climate. The long-lasting nature of human-related methane emissions underscores the importance of monitoring urban methane emissions on a yearly temporal resolution or better. By doing so, it becomes possible to track changes in methane emissions over time and measure the effectiveness of applied policies in mitigating methane emissions. By utilizing a consistent and regular monitoring approach, decision-makers and policymakers can gain a better understanding of urban methane emissions and work towards implementing effective measures to reduce their impact on the environment and climate.

Although annual monitoring is generally suitable for tracking urban methane emissions, there are situations where more frequent monitoring may be necessary. More frequent monitoring would enable the detection of seasonal variations and the identification of potential hotspots of methane emissions that may require targeted mitigation strategies. Furthermore, studies that monitor methane emissions from wetlands on a monthly basis may be necessary due to the phenology of wetland vegetation^57^. In such cases, monthly monitoring can provide insights into the intra-annual variations in methane emissions, which can help identify the main drivers of methane emissions and inform mitigation strategies. By tailoring the monitoring frequency to the specific characteristics of the methane source, decision-makers and researchers can obtain more detailed and accurate information about methane emissions^58^.

## Methodology

### Study area

**Figure 8:**
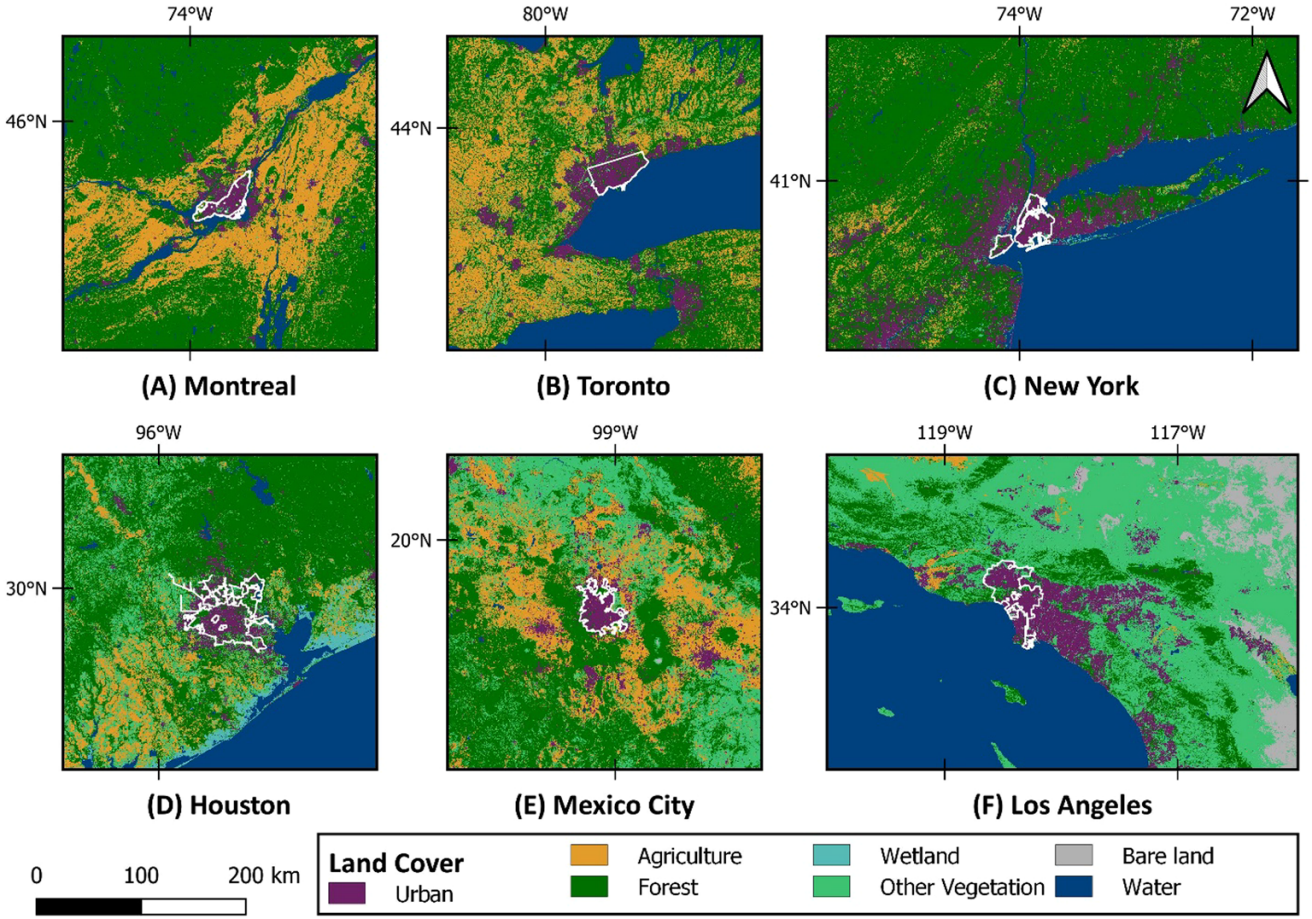
Location, boundary, and land cover map of the study area with 10 m spatial resolution from the ESA WorldCover^60^, accessed from Google Earth Engine Data Catalog (CC-BY-4.0 ). Map was created using open-source QGIS 3.34.2 (https://qgis.org/en/site/).

Covering about 16.5% of the Earth’s land area, North America has about 24 million square kilometers and a population of 579 million^6^. Canada, the United States, and Mexico are the largest and most prominent countries in the region^59^. To gain a comprehensive understanding of urban methane emissions in North America, this study focused on six metropolitan areas selected based on their population size in different countries. Specifically, the study selected three cities from the United States (i.e., New York, Los Angeles, and Houston), two from Canada (i.e., Toronto and Montreal), and Mexico City from Mexico. These six cities were chosen due to their large population sizes and representativeness of different urban contexts, allowing for a more robust analysis of urban methane emissions across the North American region. By examining methane emissions in these six metropolitan areas, this study provides valuable insights into the distribution of methane emissions and can help identify areas that may require targeted mitigation efforts to reduce their impact on the environment and climate. Figure 8 displays a map of the study area, including the land cover map of the selected metropolitan regions. As can be seen, the study area is primarily comprised of built-up land covers and urban areas. To ensure a comprehensive understanding of the total methane emissions in the metropolitan regions and their dependent facilities, this study expanded its scope beyond the city boundaries. Specifically, the study area was extended to include suburban areas that are closely linked to city-dependent activities and facilities, such as agriculture, livestock, and landfills. These extended regions are referred to as the Greater Metropolitan Areas, such as the Greater Toronto Area. By including these surrounding areas, this study provides a more complete picture of the methane emissions in each metropolitan area. Since the quantified urban methane emission reports are highly dependent on the boundaries^16^, the official cartographic boundary files of cities were accessed through the official websites or GIS services provided by the cities. Pixels that overlap the official city boundaries are used for the quantification of city emission.

### TROPOMI data

**Figure 9:**
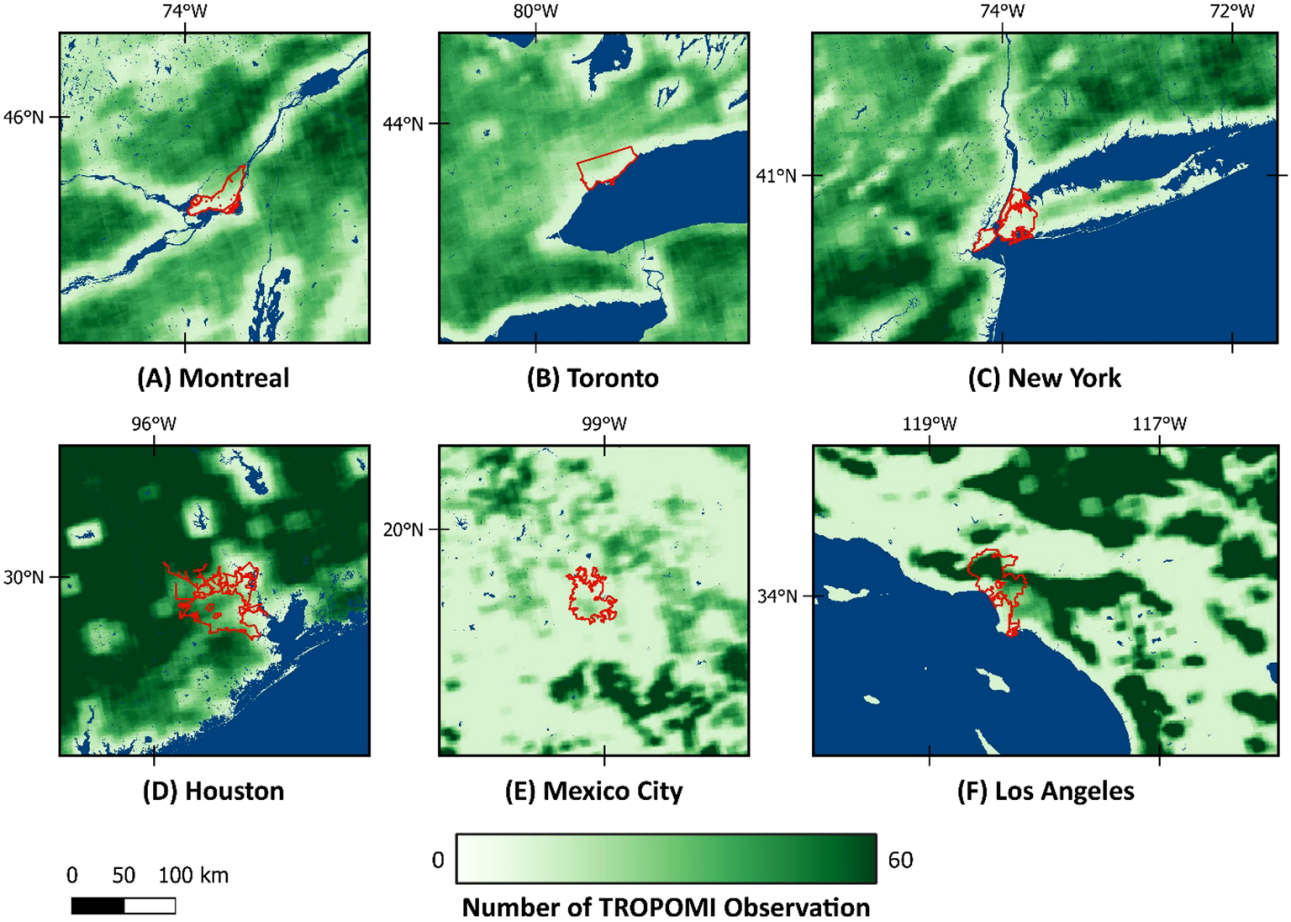
The number of TROPOMI observations available in the study area for duration of the inversion. Map was created using open-source QGIS 3.34.2 (https://qgis.org/en/site/).

In this study, we used the column-averaged dry air methane mole fractions (XCH_4_) data (Version 2.04, Level 2, algorithm version 1.2.0) produced by the Netherlands Institute for Space Research (SRON). This retrieval has near unit sensitivity down to the surface, uses the methane absorption at the shortwave infrared band (2.3 μm), and is based on the RemoTeC full-physics algorithm^61^. We accessed the TROPOMI data from 2021 on Amazon S3 (Simple Storage Service) cloud storage, which is made available by Meteorological Environmental Earth Observation S.r.l. (MEEO). In addition to XCH_4_, data includes the retrieval 12-level pressure grid, quality assurance value, vertical profile of methane dry-air mixing ratio, averaging kernel vector, boundaries, and center of the pixels, as well as surface albedo. The observation density of TROPOMI data is shown in Fig. 9. A total number of 9187 observations for Houston, 9246 for Los Angeles, 872 for Mexico City, 4447 for Montreal, 6215 for New York, and 10,648 for Toronto were used in this study. Due to the low SWIR albedo in some parts of Mexico City, the number of observations in this area was lower than in others.

**Figure 10:**
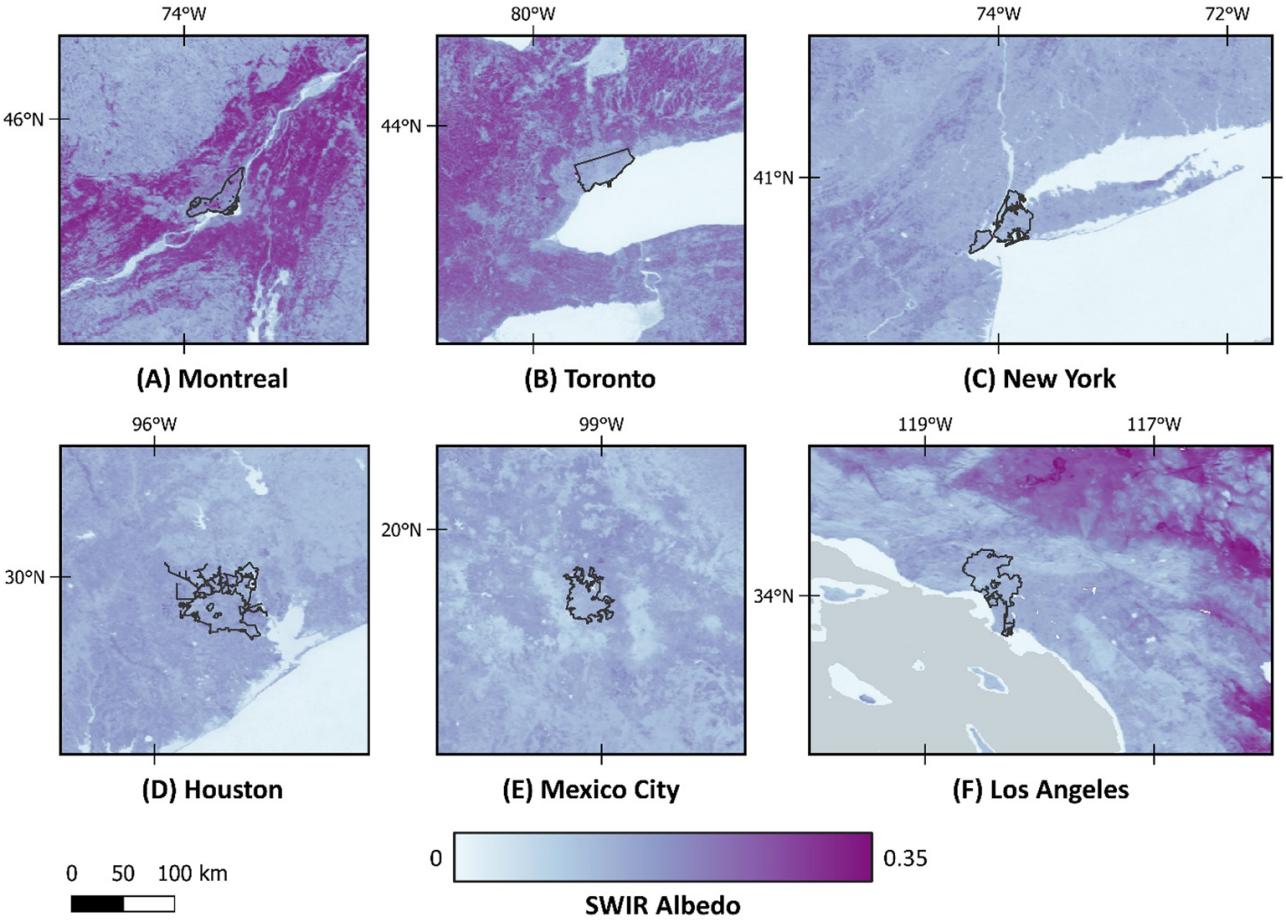
Annually-averaged SWIR albedo of the different cities in the study area. Map was created using open source QGIS 3.34.2 (https://qgis.org/en/site/).

Due to the impact of heterogeneous/dark surfaces or clouds on data quality filters, the average success rate of TROPOMI methane retrieval is 3%, only^62^. However, even with this low success rate, the global data density of TROPOMI is roughly two orders of magnitude more than GOSAT global observations over land^7^. Based on previous studies, we only used the TROPOMI methane observations over lands with a high quality (qa_value > 0.5) and above a minimum surface albedo (SWIR Albedo > 0.05). TROPOMI XCH_4_ data have a posteriori bias correction applied to reduce biases due to factors such as SWIR albedo^23^. Figure 10 shows the SWIR Albedo map in the studied region. Based on visual interpretation and comparison, no correlation between SWIR albedo and methane concentration maps was apparent. However, small albedo-dependent biases cannot be ruled out. Methods to address such biases are actively being explored (e.g., Balsus et al. 2023^63^, Lorente et al. 2023^64^), which have led to improvements in TROPOMI XCH_4_ data quality.

**Figure 11:**
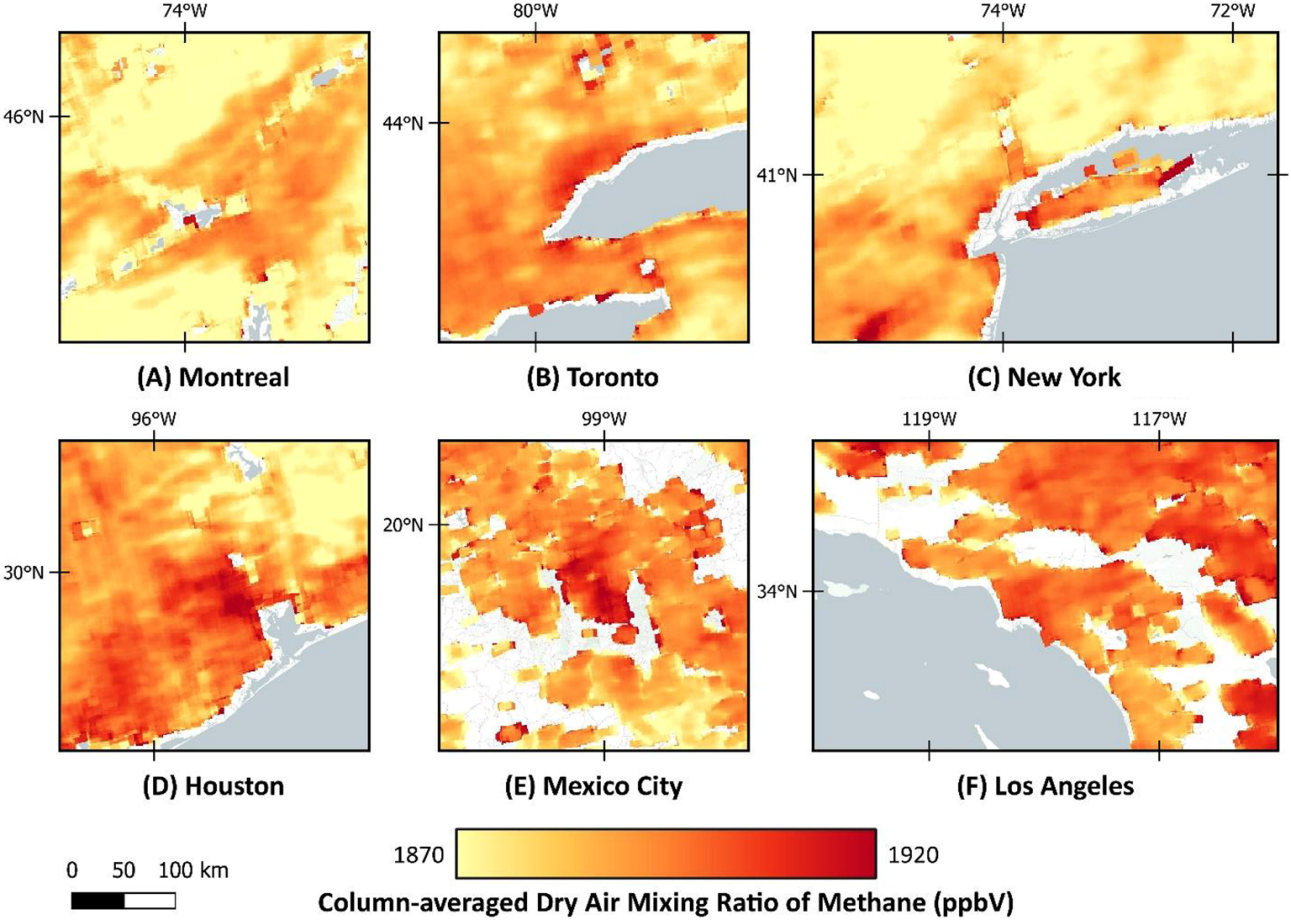
Annual-averaged value for column-averaged dry air methane ratios (XCH_4_) in 2021. Map was created using open-source QGIS 3.34.2 (https://qgis.org/en/site/).

**Figure 12:**
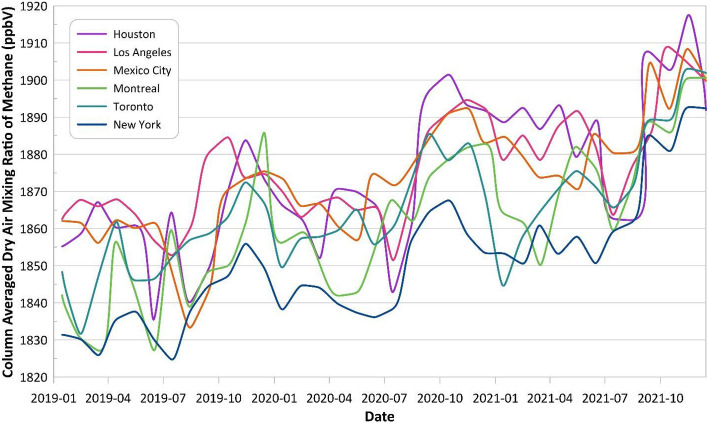
Monthly-averaged methane concentration in six big metropolitan areas in North America.

The global mean bias of TROPOMI column-averaged dry air methane ratios (XCH_4_) product is between 3 and 4 ppb when evaluated with the network of independent in-situ measurements of the Total Column Carbon Observing Network (TCCON)^23^. TCCON provides a valuable benchmark for validating satellite-derived methane measurements, and its widespread deployment allows for global comparisons of methane observations. The mean XCH_4_ for 2021 is illustrated in Fig. 11, showing hot spots over all six metropolitan areas, especially in the central parts. Figure 12 also depicts the increasing monthly-averaged methane concentration over these six cities from the beginning of 2019 to 2022, following the global trend.

### Analytical inversion

To derive methane emissions from TROPOMI data, a Chemical Transport Model (CTM) is used as the "forward model" in this analytical inversion. The CTM uses atmospheric transport and chemistry information, along with emissions data, to simulate the distribution of methane in the atmosphere. By comparing the simulated methane concentration with the observed TROPOMI data through an inversion, the system can estimate methane emissions. The use of a CTM is crucial to accurately quantify methane emissions as it takes into account the complex atmospheric processes that affect the transport and distribution of methane. In this study, we used the nested North American version of the GEOS-Chem forward model as the three-dimensional CTM to simulate the atmospheric state and methane concentration based on pre-defined emission prescription^18^. The NASA Global Modelling and Assimilation Office (GMAO) supplies the meteorological and atmospheric data from the Goddard Earth Observation System (GEOS). The GEOS data provides a comprehensive view of the Earth system, including the atmosphere, oceans, land, and their interactions. It integrates satellite observations, ground-based measurements, and numerical models to produce high-quality data products. The data are updated regularly to ensure that the model reflects the most current conditions. The 72 levels of GEOS Fast Processing (GEOS-FP)^65^ data were used, which was later merged into 47 levels with 0.25° × 0.3125° horizontal resolution. Global 4° × 5° simulations (default of IMI^47^) were used for the boundary condition. The study area was extended by a 5° buffer zone (i.e., eight buffer grid cell elements as default of IMI^47^) to create state vectors, which are the ensemble components that will be optimized in the inversion. The forward model concentration fields were initialized by using one month of spin-up simulation with prior emissions.

In order to estimate methane emissions using an analytical inversion approach, a prior estimation of the emissions is needed. These estimations are commonly referred to as emission inventories and are typically presented in a gridded format to enable integration with the inversion model. The emission inventory provides information about the distribution and amount of methane emissions from different sectors, such as agriculture, energy, and waste management. The accuracy and resolution of the emission inventory play a critical role in the accuracy of the estimated methane emission. Therefore, it is important to use the most up-to-date and accurate inventory available, which can be challenging due to the dynamic nature of emissions and the varying quality and availability of data across different regions. Nevertheless, the use of emission inventories remains a critical step in methane monitoring and mitigation efforts.

Emission inventories used in this study, all available in a gridded format from IMI cloud-platform^47^, are listed in Table 3. As reported to the United Nations Framework Convention on Climate Change (UNFCCC), we used U.S.^49^, Canada^50^, and Mexico^51^ sector-resolved methane emission inventory for all the anthropogenic methane emission sectors, including fuel exploitation (i.e., coal, oil, and gas), livestock, landfills, and wastewater. For natural methane emissions, we used monthly wetland emissions, open fires, and a very small contribution from termites and geological seeps.

**Table 3:** Emission bottom-up inventories for different sectors, accessed from IMI platform^47^.

Sector	Inventory
Anthropogenic (gridded version)	Spatially-distributed gridded inventory of the U.S. emissions based on the Environmental Protection Agency (EPA) Greenhouse Gas Inventory (GHGI) Inventory of US Greenhouse Gas Emissions and Sinks^49^ for 2012
	Spatially-distributed gridded inventory of Canadian emissions based on the Environment and Climate Change Canada (ECCC) National Inventory Report for Canada^50^ for 2018
	Spatially-distributed gridded inventory of Mexico’s emissions based on the National Institute of Ecology and Climate Change (INECC) national inventory^51^ for 2015
Wetlands	Wetland Methane Emissions and Uncertainty^58^ (WetCHARTs v1.3.1)
Geological seeps	Geological methane emissions and their isotopic signature^66^
Open fires	Global Fire Emissions Database^67^ (GFED4)
Termites	Three-dimensional model synthesis of the global methane cycle^68^

The forward model (GEOS-Chem) simulation is fitted to the TROPOMI observations to infer methane emissions using Bayesian inverse analysis. This includes setting weights to prior estimations and related uncertainties, assuming a probability density function with normal error, and minimizing the scalar cost function (Eq. 1)^48^.

1 $${J}_{(x)}={\left(x-{x}_{A}\right)}^{T}{S}_{A}^{-1}\left(x-{x}_{A}\right)+\gamma {\left(y-{K}_{x}\right)}^{T}{S}_{O}^{-1}\left(y-{K}_{x}\right),$$

In this cost function, ***x*** is the emission state vector, and ***x***_*A*_ is the prior emission, estimated in the emission inventories. Jacobian matrix (***K***_***x***_) indicates the sensitivity of column-averaged methane concentrations (XCH_4_) from TROPOMI (***y***) to perturbation of emissions, as described by the GEOS-Chem simulation. ***S***_***O***_ is the covariance matrix for TROPOMI observational error and was assumed to be 15 ppb based on default IMI value^47^. ***S***_***A***_ is the covariance matrix for prior emissions and γ is the regularization factor. To include errors from the inversion parameters, a grid search was done based on the range of ***S***_***A***_ and γ and also the suggested values from default IMI^47^, and previous studies^1,32^. As a result of this grid search, an ensemble inversion was created with different combinations of inversion parameters, and the average values were reported for emissions.

2 $$\widehat{x}= {x}_{A}+ {(\gamma {K}^{T}{S}_{O}^{-1}K+ {S}_{A}^{-1})}^{-1} \gamma {K}^{T}{S}_{O}^{-1}\left(y-K{x}_{A}\right),$$

3 $$\widehat{S}= {(\gamma {K}^{T}{S}_{O}^{-1}K+ {S}_{A}^{-1})}^{-1},$$

4 $$A= {I}_{n}-\widehat{S}{S}_{A}^{-1},$$

By analytical solution of ${\nabla }_{x}{J}_{(x)}=0$, we obtained the posterior optimal estimate (Eq. 2), the error covariance matrix for the posterior estimate (Eq. 3), and the matrix of the averaging kernel (Eq. 4) for each ensemble member. The sensitivity of $\widehat{x}$ to the actual values ($\frac{\partial \widehat{x}}{\partial x}$) is described by the averaging kernel matrix, and the degrees of freedom is the trace of this matrix. We used the Integrated Methane Inversion (IMI V1.0) cloud-computing tool to infer the methane emissions using analytical inversion^47^. The IMI framework takes advantage of the Amazon Web Services (AWS) cloud computing platform to access appropriate computation resources relative to the time frame and scale of the inversion^47^. In addition, this framework enhances data management by using the available TROPOMI and GEOS-Chem chemical transport models on AWS. Considering the time and scale of the inversion, we used an AWS EC2 Linux virtual machine (c5.12xlarge) with 48 vCPU and 96 Gigabytes of memory. The results were produced for cities individually, and then the results were merged together in the post-processing.

## Conclusion

Methane monitoring is crucial for mitigating the impact of climate change as methane is a potent greenhouse gas with a higher global warming potential than carbon dioxide. Accurately measuring and tracking methane emissions is a real need for developing effective policies and techniques to reduce its environmental impact. The use of satellite data for methane monitoring offers a scalable approach for estimating emissions at various scales, which is essential for effective climate change mitigation efforts. The feasibility of TROPOMI observations and the IMI tool^47^ for inferring methane emissions at an urban scale through an atmospheric inversion model has been investigated in this study. Considering the daily and global observations of TROPOMI, only a limited number of them are suitable for use in the inversion, and a majority of observations are low quality or are filtered due to cloud coverage and snow. In this study, we used one year of TROPOMI observations in 2021, along with atmospheric simulations of the GEOS-Chem chemical transport model through the IMI platform^47^. This simulation is based on meteorological data from NASA and needs an estimation of prior emissions in the study area. To obtain the best knowledge available from the emission inventories, we used the gridded national and global estimations of anthropogenic and natural methane fluxes available from the IMI tool^47^ in this study.

We used the analytical solution to infer methane emission through the Bayesian inversion analysis from the IMI^47^ to quantify methane emissions across North American urban areas. Scale factors resulting from the analytical inversion indicate the correction factor needs to be applied to the prior estimates of the emissions, and the reported bottom-up inventories had an underestimation of methane emission in most cities. Utilizing the outcomes of ensemble inversions and considering city boundaries, the mean annual total emissions were recorded as Toronto at 230.52 Gg a^−1^, Montreal at 111.54 Gg a^−1^, New York at 144.38 Gg a^−1^, Los Angeles at 207.03 Gg a^−1^, Houston at 650.16 Gg a^−1^, and Mexico City at 280.81 Gg a^−1^. Scale factors in this study ranged from 0.22 to 6.2, showing the variation of under-estimations and overestimation of prior methane in the study areas. To analyze the confidence of the results, an ensemble inversion was performed, and inversion parameters were tuned based on suggested values of previous TROPOMI studies and a grid search. Continued efforts to monitor and track methane emissions will be crucial in mitigating the impact of this potent greenhouse gas on the environment and achieving a sustainable future.

## Data Availability

TROPOMI data can be accessed via Copernicus Data Space Ecosystem: https://dataspace.copernicus.eu/ (Accessed March 20, 2024). GEOS-Chem CTM and related datasets can be accessed via: https://geoschem.github.io/ (Accessed March 20, 2024). Integrated Methane Inversion (IMI), including related documents and AWS Marketplace links, can be accessed via: https://imi.seas.harvard.edu/ (Accessed March 20, 2024).
